# Anti-Inflammatory Comparison of Melatonin and Its Bromobenzoylamide Derivatives in Lipopolysaccharide (LPS)-Induced RAW 264.7 Cells and Croton Oil-Induced Mice Ear Edema

**DOI:** 10.3390/molecules26144285

**Published:** 2021-07-15

**Authors:** Pimpichaya Sangchart, Panyada Panyatip, Teerasak Damrongrungruang, Aroonsri Priprem, Pramote Mahakunakorn, Ploenthip Puthongking

**Affiliations:** 1 Graduate School, Khon Kaen University, Khon Kaen 40002, Thailand; pimpichaya@kkumail.com; 2 Melatonin Research Group, Faculty of Pharmaceutical Sciences, Khon Kaen University, Khon Kaen 40002, Thailand; ppanyada90@gmail.com (P.P.); dteera@kku.ac.th (T.D.); pramah@kku.ac.th (P.M.); 3 Division of Oral Diagnosis, Department of Oral Biomedical Sciences, Faculty of Dentistry, Khon Kaen University, Khon Kaen 40002, Thailand; 4 Faculty of Pharmacy, Mahasarakham University, Maha Sarakham 44150, Thailand; aroonsri@kku.ac.th; 5 Department of Pharmacognosy and Toxicology, Faculty of Pharmaceutical Sciences, Khon Kaen University, Khon Kaen 40002, Thailand; 6 Department of Pharmaceutical Chemistry, Faculty of Pharmaceutical Sciences, Khon Kaen University, Khon Kaen 40002, Thailand

**Keywords:** melatonin, amide derivative, anti-inflammation, RAW 264.7 cells, LPS, mice ear edema

## Abstract

The pineal gland is a neuroendocrine organ that plays an important role in anti-inflammation through the hormone melatonin. The anti-inflammatory effects of melatonin and its derivatives have been reported in both in vitro and in vivo models. Our previous study reported the potent antioxidant and neuroprotective activities of bromobenzoylamide substituted melatonin. In silico analysis successfully predicted that melatonin bromobenzoylamid derivatives were protected from metabolism by CYP2A1, which is a key enzyme of the melatonin metabolism process. Therefore, the anti-inflammatory activities of melatonin and its bromobenzoylamide derivatives BBM and EBM were investigated in LPS-induced RAW 264.7 macrophages and croton oil-induced ear edema in mice. The experiments showed that BBM and EBM significantly reduced production of the inflammatory mediators interleukin-6 (IL-6), prostaglandin E2 (PGE_2_), and nitric oxide (NO) in a dose-dependent manner, but only slightly affected TNF-α in LPS-induced RAW 264.7 macrophages. This suggests that modifying melatonin at either the *N1*-position or the *N*-acetyl side chain affected production of NO, PGE_2_ and IL-6 in in vitro model. In the croton oil-induced mouse ear edema model, BBM, significantly decreased ear edema thickness at 2–4 h. It leads to conclude that bromobenzoylamide derivatives of melatonin may be one of the potential candidates for a new type of anti-inflammatory agent.

## 1. Introduction

Inflammation is a defense mechanism against pathogens. It is a complex process involving multiple cell types and mediators. Macrophages play an important role in the inflammatory response to pathogens by releasing pro-inflammatory mediators, including tumor necrosis factor-α (TNF-α), interleukin (IL)-1β, IL-6, cyclooxygenase (COX)-2, and nitric oxide (NO) [1]. Overproduction of these mediators and non-resolution of inflammation leads to the pathophysiology of many chronic inflammatory diseases, such as rheumatoid arthritis (RA), systemic lupus erythematosus (SLE), chronic obstructive pulmonary disease (COPD), Crohn’s disease, ulcerative colitis and some neurodegenerative diseases [2].

**Figure 1:**
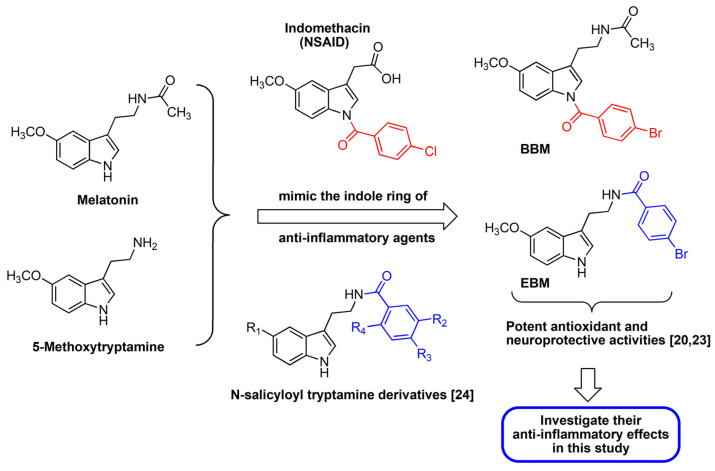
Design of the target compounds; BBM and EBM.

Melatonin and its metabolites, *N1*-acetyl-*N2*-formyl-5-methoxykynuramine (AFMK) and *N1*-acetyl-5-methoxykynuramine (AMK), have antioxidant and anti-inflammatory activities. They are highly efficient NO scavengers [3,4,5,6], and melatonin has been shown to reduce the production of pro-inflammatory cytokines such as interferon gamma (IF-γ), TNF-α, IL-1β, and IL-6, inhibit NO production, and suppress inducible nitric oxide synthase (iNOS) and COX-2 gene expression [4,7,8]. Melatonin has also been shown to exhibit anti-inflammatory effects in vivo [9,10,11]. Although melatonin has low toxicity, its usefulness is limited by its short half-life and low oral bioavailability [12,13,14,15]. Therefore, several synthetic melatonin derivatives that retain melatonin’s bioactivities but improve upon these limitations have been developed [7,16,17,18,19]. *N*-amide melatonin derivatives that mimic the indole ring residue of Non-Steroidal Anti-Inflammatory Drugs (NSAIDs) were synthesized by our group (Figure 1). The derivatives with bromobenzoylamide-substitutions had higher antioxidant capacity than unsubstituted derivatives, and this affected the electron spin resonance (ESR) signal [20]. This result is supported by the study of Mor et al. who showed that increasing lipophilicity at the indole ring of melatonin improved antioxidant activity [21]. It is well known that inflammation processes are related to increases in free radicals such as reactive oxygen species (ROS) [6].

Interestingly, an in silico study predicted that the *N1*-position lipophilic-substituted melatonin derivative (*N*-(2-(1-4-bromobenzoyl-5-methoxy-1H-indol-3-yl)ethyl) acetamide, BBM) could not be metabolized by CYP1A2, which prolonged its half-life compared with melatonin [22]. In contrast, while another melatonin derivative with a 4-bromobenzoyl amide substitution at the *N*-acyl side chain (4-bromo-*N*-(2-(5-methoxy-1H-indol-3-yl)ethyl)benzamide, EBM) was predicted to be a substrate of CYP1A2, it displayed a potent antioxidant and neuroprotective effect [20,23]. Fan et al. [24] also reported that *N*-salicyloyl tryptamine derivatives are potential anti-neuroinflammation agents that act by decreasing the production of proinflammatory mediators, NO, PGE_2_ and TNF-α. These considerations prompted us to investigate and evaluate the anti-inflammatory activity of the BBM and EBM lipophilic melatonin derivatives. The present study compared the inhibitory effects of BBM and EBM with melatonin on inflammation mediators such as NO, PGE_2_, IL-6 and TNF-α in LPS-induced RAW 264.7 macrophages and on acute inflammation in vivo in croton oil-induced mouse ear edema. The synthesis and structure elucidation of BBM and EBM have been previously described [20]. The molecular information is shown in the Supplementary Material (Figures S1–S6).

## 2. Results

### 2.1. Effect of Melatonin and Its Derivatives on the Viability of RAW264.7 Cells

**Figure 2:**
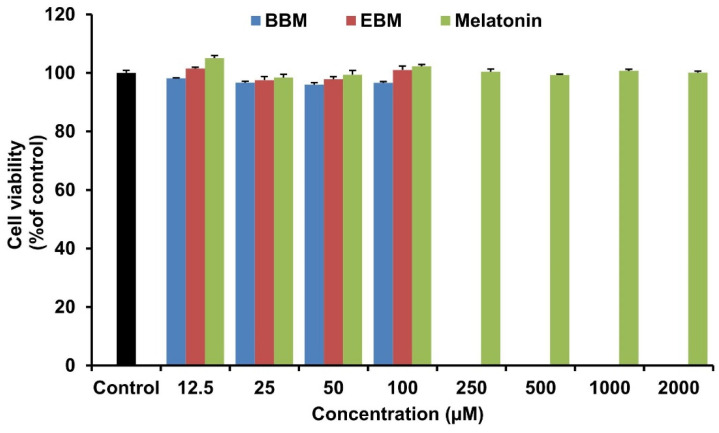
Viability of RAW 264.7 cells after treatment with melatonin, BBM, and EBM in various concentrations. The percentage cell viabilities were calculated relative to viability of the vehicle-treatment group. Values are expressed as means ± S.E.M (n = 8).

To determine the toxicity of the tested compounds, RAW 264.7 cells were treated with melatonin and its derivatives. The cells were incubated with 12.5–2000 µM BBM, EDM or melatonin for 24 h, and cell viability was determined by MTT assay. The results show that cell viability was above 80% for BBM and EBM in the concentration range 12.5–100 µM, while melatonin did not affect cell viability up to 2000 µM (Figure 2). According to the ISO 10993-5, cell viability of more than 80% is acceptable to be non-toxic [25]. Therefore, all compounds were considered non-toxic to RAW 264.7 cells in the range of concentrations used in this present study.

### 2.2. Effect of Melatonin and Its Derivatives on NO Production in LPS-Stimulated RAW264.7 Cells

**Figure 3:**
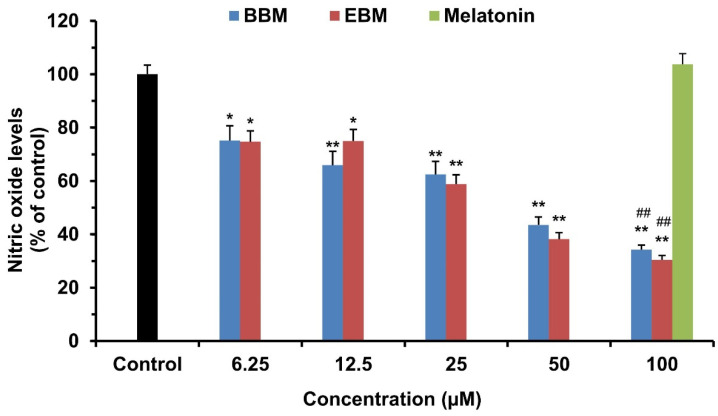
Effect of melatonin, BBM and EBM on the production of nitric oxide in LPS-stimulated RAW 264.7 cells. Values are expressed as the means ± S.E.M * *p* < 0.05, ** *p* < 0.001 versus vehicle treatment group, ## *p* < 0.001 versus melatonin 100 µM.

The production of NO was determined from the culture medium of LPS-stimulated RAW 264.7 cells. Cells were treated simultaneously with LPS and various concentrations of melatonin or its derivatives, BBM and EBM, for 24 h. The production of NO was determined by Griess reagent. As shown in Figure 3, BBM and EBM inhibited LPS-induced NO production by RAW 264.7 cells at all tested concentrations (6.25–100 μM) in a concentration dependent manner. Both modified derivatives had significantly higher potency in inhibiting the production of NO than melatonin, which suggests that NO production was influenced by the lipophilic substituents at both the *N1*- and *N*-acyl side chain positions. This was supported by the IC_50_ values of BBM, EBM, and MLT, which were 39.81, 34.52, and >100 µM, respectively.

### 2.3. Effect of Melatonin and Its Derivatives on PGE_2_ Production in LPS-Stimulated RAW264.7 Cells

**Figure 4:**
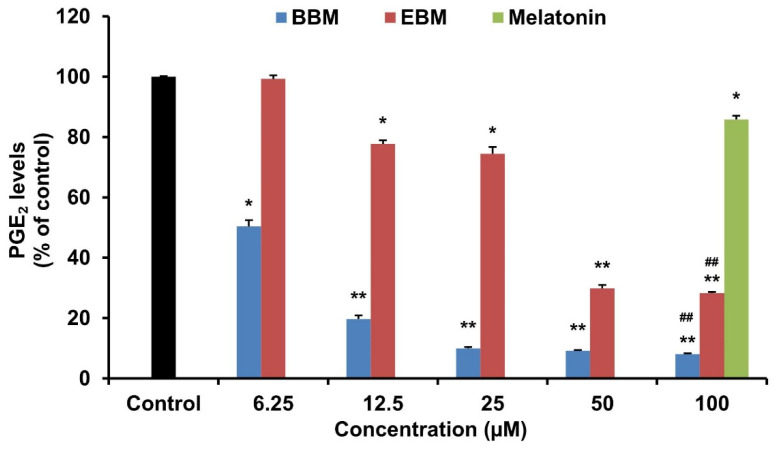
Effect of melatonin, BBM and EBM on the production of PGE_2_ in LPS-stimulated RAW 264.7 cells. Values are expressed as the means ± S.E.M * *p* < 0.05, ** *p* < 0.001 versus vehicle treatment group, ## *p* < 0.001 versus melatonin 100 µM.

In LPS-induced RAW246.7 macrophages, BBM and EBM significantly (*p* < 0.05) decreased the production of the proinflammatory mediator PGE_2_, at 6.25 and 12.5 µM, respectively (Figure 4). A comparison between melatonin and its modified derivatives at the same concentration (100 µM) shows that the BBM and EBM derivatives had a higher inhibitory effect on the production of PGE_2_ than melatonin. Among the two derivatives, BBM exhibited the best activity with an IC_50_ value of 7.02 µM, whereas the EBM and melatonin IC_50_ values were 35.20 and >100 µM, respectively.

### 2.4. Effect of Melatonin and Its Derivatives on IL-6 and TNF-α Production in LPS-Stimulated RAW264.7 Cells

**Figure 5:**
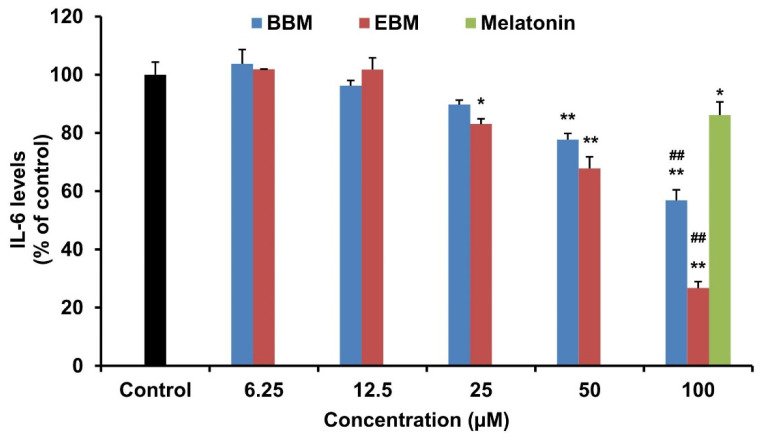
Effect of melatonin, BBM and EBM on the production of IL-6 in LPS-stimulated RAW 264.7 cells. Values are expressed as the means ± S.E.M * *p* < 0.05, ** *p* < 0.001 versus vehicle treatment group, ## *p* < 0.001 versus melatonin 100 µM.

**Figure 6:**
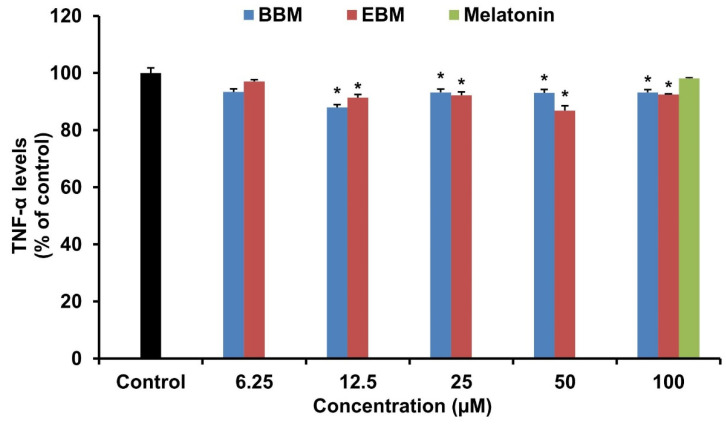
Effect of BBM and EBM on the production of TNF-α in LPS-stimulated RAW 264.7 cells. Values are expressed as the means ± S.E.M * *p* < 0.05 versus vehicle treatment group.

The effect of melatonin and its derivatives on the production of pro-inflammatory cytokines, IL-6 and TNF-α were investigated in LPS-stimulated RAW 264.7 cells. LPS-induced IL-6 production was significantly suppressed by EBM from 25 µM, BBM from 50 µM and melatonin at 100 µM (Figure 5). Both derivatives reduced the level of IL-6 by more than their parent compound at 100 µM. EBM established the highest activity with an IC_50_ value of 71.03 µM, while melatonin and BBM both had IC_50_ values > 100 µM. Melatonin and its derivatives did not alter TNF-α levels in LPS-stimulated RAW 264.7 cells (Figure 6).

### 2.5. In Vivo Anti-Inflammatory Activity

Croton oil-induced ear edema is an in vivo model widely used for investigation of topical acute anti-inflammatory activity. This model is rapid and simple. Moreover, it requires only a small amount of substance to perform this method [26]. Topical application of all tested compounds significantly decreased ear thickness at 1–4 h after exposure to croton oil. Aspirin, the positive control, inhibited mice ear edema by 63.6 ± 5.8% at 1 h after application of croton oil and reached peak inhibition of 88.2 ± 7.4% at 3 h (Table 1). Melatonin inhibited ear edema by a maximum of 76.9 ± 10.3% at 4 h after exposure to croton oil. BBM inhibited edema by 65.0 ± 12.0% after 2 h and 76.9 ± 10.3% inhibition at 4 h. Peak inhibition of ear edema by EBM was 69.2 ± 9.7% at 4 h.

## 3. Discussion

Macrophages are effector cells which play an important role in inflammatory processes. Activated macrophages produce pro-inflammatory cytokines such as IL-1β, IL-6, and TNF-α, as well as PGE_2_ and NO [27]. RAW 264.7 murine macrophage cells have been widely used for in vitro anti-inflammatory studies. These cells are sensitive to LPS stimulation, which is an endotoxin isolated from gram negative bacteria [28]. LPS activates the nuclear factor kappa B (NF-κB) pathway, thus releasing pro-inflammatory cytokines [29]. According to the current study, RAW 264.7 macrophages exposed to LPS produced nitrite, which implied NO production. Our results show that NO production was significantly inhibited by BBM and EBM in a dose-dependent manner at concentrations as low as 6.25 μM. BBM and EBM exhibited significantly higher potency than melatonin at 100 μM. Interestingly, both the modified melatonin structures with the lipophilic substitutions at either the *N1*-indole ring or the *N*-acyl side chain are better NO suppressors than the parent structure melatonin. It has been reported that anti-inflammatory actions of melatonin depend on the inhibition of the expression of iNOS and the efficiency of NO scavenging [5].

In addition to inhibition of NO production, melatonin and its derivatives also significantly inhibited the production of PGE_2_ and IL-6. Furthermore, both derivatives demonstrated higher potency than melatonin at the same concentration (100 μM). This result correlates with previous reports that melatonin inhibited the expression of iNOS and COX-2, both of which are key enzymes that catalyze the production of NO and PGEs [30,31,32,33]. Among the derivatives, BBM displayed the highest inhibitory effect on PGE_2_ production, and EBM had the highest inhibitory effect on IL-6. A previous report showed that the effect of modifying the *N*-acetyl side chain of melatonin was to inhibit the production of NO, TNF-α, IL-6, and pro-IL-1β in LPS-induced RAW 264.7 cells [7]. Taken together, this suggests that the production of NO, PGE_2_ and IL-6 are affected by modification at either the *N1*-position or the *N*-acetyl side chain of melatonin. In contrast, both derivatives only slightly affected TNF-α production.

NO, PGE_2_ and IL-6 are considered to be the most important mediators of inflammation both in vitro and in vivo [34,35]. Therefore, we employed the in vivo acute anti-inflammatory model using a croton oil model to induce acute inflammation for investigation of topical anti-inflammatory agents. 12-O-tetradecanoylphorbol-13-acetate (TPA), the main irritant contained in croton oil, activates protein kinase C, which stimulates the release of pro-inflammatory cytokines including IL-1β, TNF-α and IL-6, and other mediators such as phospholipase A2 and arachidonic acid [36]. Leukotrienes and prostaglandins are arachidonic acid metabolites produced by 5-LOX and COX enzymes [37]. Shin et al. [38] indicated that application of croton oil increased expression of COX-1 and COX-2 and increased PGE_2_ production. These mechanisms increase vascular permeability, vasodilation and swelling due to the release of histamine and serotonin, which is followed by the synthesis of leukotrienes and prostaglandins [39]. Therefore, anti-inflammatory drugs such as aspirin that inhibit COX enzymes are effective in suppressing ear edema induced by croton oil [40,41]. Ear edema induced by croton oil peaks about 6 h after exposure [42]. Priprem et al. [43] reported that application of a melatonin gel reduced mouse ear edema 1 h after croton oil stimulation. Therefore, the present study decided to evaluate inhibition of ear edema after application of croton oil at 1–4 h. The results showed that all tested compounds reduced mice ear edema volumes, measured as ear thickness. BBM showed a significantly reduced ear thickness compared to melatonin at 2–4 h. Melatonin and BBM inhibited croton oil induced mice ear edema and reached a plateau of inhibition at 3–4 h, which was similar to aspirin. Interestingly, BBM dramatically inhibited mice ear edema (65% inhibition) at 2 h, while melatonin and EBM reached the edema inhibition > 65% at 3 and 4 h, respectively. It is well-known that the lipophilic molecules are the preferred passive skin permeation. As results of in silico predictions (Supplementary Materials; Table S1), BBM was characterized as a high skin permeation (log *K*_p_; −6.15) [44]. The presence of the bromobenzoylamide group at the *N1*-position of the melatonin structure could increase the lipophilicity (log *P*_o/w_ 3.36) and topological polar surface area (TPSA; 60.33 A^0^), which are the key parameters for absorption prediction [45].

The modified compound, BBM, showed similar acute anti-inflammatory effects to melatonin in vivo, but had greater NO, IL-6 and PGE_2_ suppressing effects in vitro. The rate-determining step of partitioning into the stratum corneum depends on the lipophilicity of the permeant molecules, whereas partitioning from the stratum corneum into the viable epidermis and dermis involves other physicochemical parameters, including solubility and dissociation, in addition to lipophilicity [44]. Despite similarities in the permeability coefficients of melatonin and BBM, the increased lipophilicity, molecular size and decreased water solubility of BBM potentially alters transport through the skin, affecting in vivo anti-inflammatory activity but not in vitro. Additionally, the anti-inflammatory effects of melatonin involve several pathways, 5-LOX, iNOS and COX-2 [5,8,46,47] that should be thoroughly explored. Further investigations are essential to develop the potential applications of melatonin derivatives.

## 4. Materials and Methods

### 4.1. Chemicals

Melatonin was purchased from Shanghai Chemical Co. Ltd. (Shanghai, China). The melatonin derivatives, BBM and EBM, were synthesized and characterized by the Melatonin Research Group, Khon Kaen University, Thailand [20]. RPMI 1640, fetal bovine serum (FBS), antibiotic-antimycotic (100X), 5% trypsin-EDTA, Dulbecco’s phosphate-buffered saline (DPBS) 10X, and 0.4% trypan blue stain were purchased from Gibco. Inc. (New York, NY, USA). Further, 3-(4, 5-dimethyl-2-thiazolyl)-2, 5-diphenyl-2H-tetrazolium bromide (MTT) was purchased from Invitrogen (Eugene, Oregon, USA). Lipopolysaccharides (LPS) from *Escherichia coli* 0111:B4, sulfanilamide and naphthylethylenediamine dihydrochloride (NED) were purchased from Sigma-Aldrich (St. Louis, MO, USA). Dimethyl sulfoxide (DMSO), and phosphoric acid and acetone were obtained from Fisher Chemical (Loughborough, UK). Mouse PGE_2_, IL-6, TNF-α ELISA kits were purchased from R&D Systems, A Bio-Techne (Minneapolis, MN, USA). Aspirin and croton oil were purchased from Sigma-Aldrich (St. Louis, MO, USA).

### 4.2. Cell Culture

The murine macrophage cells, RAW 264.7, were obtained from Cell Lines Service (Eppelheim, Germany) and cultured in RPMI 1640 medium supplemented with 10% FBS and 1% antibiotic-antimycotic. Cells were incubated at 37 °C and humidified with 5% CO_2_ conditions. The medium culture was replaced every 2–3 days until the cells reached 80% confluence. Cells were counted by hemocytometer with Trypan blue staining.

### 4.3. Cytotoxicity

RAW 264.7 cells were seeded in 96-well plates at a density of 2 × 10^4^ cells/well and incubated for 24 h. The culture medium was then changed, and cells were treated with melatonin (12.5–2000 µM), BBM or EBM (12.5–100 µM) at concentrations dependent on their solubility, and incubated for another 24 h. Cell viability was measured by MTT assay by incubation with 0.5 mg/mL of MTT solution for 4 h. Formasan crystals were dissolved by DMSO, and the absorbance was determined at 550 nm by microplate reader (Ensight, Perkin Elmer Inc., Waltham, MA, USA). Cell viability was calculated, and the non-toxic concentrations were used in further experiments.

### 4.4. In Vitro Anti-Inflammatory Activity

#### 4.4.1. Determination of NO Production

Nitrite accumulation was measured as an indicator of NO production. RAW 264.7 cells were seeded in 24-well plates for 24 h prior to incubation with melatonin, BBM or EBM in the presence of 1 µg/mL of LPS. After 24 h of incubation, the supernatant of cultured medium was collected to determine the NO production. The quantity of nitrite was measured as an indicator of NO production by using Griess reagent at the absorbance at 550 nm. The Griess reagent was a mixture of 2% (*w/v*) of sulfanilamide and 0.2% (*w/v*) of NED in 2.5% phosphoric acid. The quantity of nitrite was determined from a sodium nitrite standard curve.

#### 4.4.2. Determination of PGE_2_, IL-6, and TNF-α Levels

The supernatants of cultured medium were collected in the same way as the NO experiment. After stimulation with LPS and incubation with melatonin or its derivatives, supernatants of RAW 264.7 cells were collected to measure the PGE_2_, IL-6, or TNF-α levels by commercial ELISA kits following the manufacturer’s instructions.

### 4.5. In Vivo Anti-Inflammatory Activity

#### 4.5.1. Animals

Nine to twelve-week-old male ICR mice, weight 25–30 g, were purchased from Nomura Siam International Co, Ltd., Bangkok, Thailand. The mice were housed in groups of 6 per cage, with access to pellet food and water. The room was maintained at a 12 h light–dark cycle, and was 23 ± 2 °C temperature controlled.

#### 4.5.2. Croton Oil-Induced Ear Edema

Mice were randomly assigned into 6 per group. Melatonin, BBM or EBM solutions were prepared as 1% (*w/v*) in acetone. Ten µL of melatonin or its derivatives (100 µg/ear) was applied to the left outer ears of the mice 30 min prior to applying 5% croton oil to the left inner ears. The thickness of ears was measured every 1 h to 4 h. The inhibition of ear edema was calculated compared to the initial thickness. Acetone and 5% aspirin were used as negative and positive controls, respectively. The percentage of inhibition was calculated by the following Equation (1).

% Inhibition of ear edema = [(∆_Neg CTR_ − ∆_Sample_)/∆_Neg CTR_] × 100 (1)

where ∆_Neg CTR_ = different of ear thickness at time_n_ and initial time of negative control group, ∆_Sample_ = different of ear thickness at time_n_ and initial time of sample group.

### 4.6. Statistical Analysis

Data are expressed as mean ± standard error of mean (S.E.M). The statistics were analyzed using the one-way analysis of variance (ANOVA) followed by Tukey’s post hoc test. The *p*-values less than 0.05 (*p* < 0.05) were considered statistically significant. The comparisons of ear thickness and inhibition of ear edema were conducted using the independent *t*-test. *p* < 0.05 indicated statistically significant differences.

## 5. Conclusions

Two melatonin derivatives modified at the *N1*-position and the *N*-acetyl side chain exhibited superior inhibition of the production of NO, PGE_2_ and IL-6 in LPS-induced inflammatory responses in macrophage RAW 264.7 cells compared to their parent melatonin. The 4-bromobenzoyl *N*-substituted modified melatonin derivatives showed the same acute anti-inflammatory effect as melatonin in a croton oil-induced mouse ear edema model, but with improved pharmacokinetic parameters. Therefore, BBM appears to be a promising new anti-inflammatory agent, and further studies into its mechanism of action and pharmacokinetic properties are warranted.

## Figures and Tables

**Table 1:** Ear thickness of mice (mm) and % inhibition of croton oil-induced ear edema.

Time (h)	Control Group	Aspirin		BBM		EBM		Melatonin	
	Thickness (mm)	Thickness (mm)	% Inhibition	Thickness (mm)	% Inhibition	Thickness (mm)	% Inhibition	Thickness (mm)	% Inhibition
Initial	0.192 ± 0.005	0.193 ± 0.003	-	0.180 ± 0.004	-	0.183 ± 0.006	-	0.197 ± 0.006	-
1	0.228 ± 0.005	0.207 ± 0.002 ^a^	63.6 ± 5.8 ^a^	0.207 ± 0.003 ^a^	27.3 ± 9.1 ^b^	0.205 ± 0.003 ^a^	40.9 ± 10.9 ^a^	0.218 ± 0.006	40.9 ± 4.6 ^a,b^
2	0.225 ± 0.004	0.207 ± 0.005 ^a^	60.0 ± 6.3 ^a^	0.192 ± 0.004 ^a,b,c^	65.0 ± 12.0 ^a^	0.200 ± 0.005 ^a^	50.0 ± 10.0 ^a^	0.217 ± 0.006	40.0 ± 11.0 ^a^
3	0.220 ± 0.003	0.197 ± 0.004 ^a^	88.2 ± 7.4 ^a^	0.187 ± 0.004 ^a,c^	76.5 ± 7.4 ^a^	0.197 ± 0.007 ^a^	52.9 ± 7.4 ^a,b^	0.203 ± 0.006 ^a^	76.5 ± 11.8 ^a^
4	0.213 ± 0.003	0.197 ± 0.004 ^a^	84.6 ± 9.7 ^a^	0.185 ± 0.004 ^a,b,c^	76.9 ± 10.3 ^a^	0.190 ± 0.005 ^a^	69.2 ± 9.7 ^a^	0.202 ± 0.005 ^a^	76.9 ± 10.3 ^a^

Note: ^a,b,c^ *p* < 0.05 versus no-treatment (control group), aspirin-treated and melatonin-treated groups, respectively.

## Data Availability

The data presented in this study are available on request from the corresponding author.

## Supplementary Materials

The following are available online. Figure S1. ^1^H-NMR spectrum of BBM. Figure S2. ^13^C-NMR spectrum of BBM. Figure S3. IR spectrum of BBM. Figure S4. ^1^H-NMR spectrum of EBM. Figure S5. ^13^C-NMR spectrum of EBM. Figure S6. IR spectrum of EBM, Table S1. MW, Lipophilicity values in Octanol-water (Log Po/w), topological polar surface area (TPSA), solubility parameter (log *S*), permeability coefficient (log *K*p) predicted from Swiss ADME.

## Sample Availability

Samples of melatonin and its derivatives are available from the authors.
