# Supplementary material for: Anti-Inflammatory Comparison of Melatonin and Its Bromobenzoylamide Derivatives in Lipopolysaccharide (LPS)-Induced RAW 264.7 Cells and Croton Oil-Induced Mice Ear Edema
Source: Molecules. 2021 Jul 15;26(14):4285. doi: 10.3390/molecules26144285 (PMC8304993; doi:10.3390/molecules26144285)
Supplement: Supplementary file 1 [file molecules-26-04285-s001.zip › molecules-1261519-supplementary.pdf]

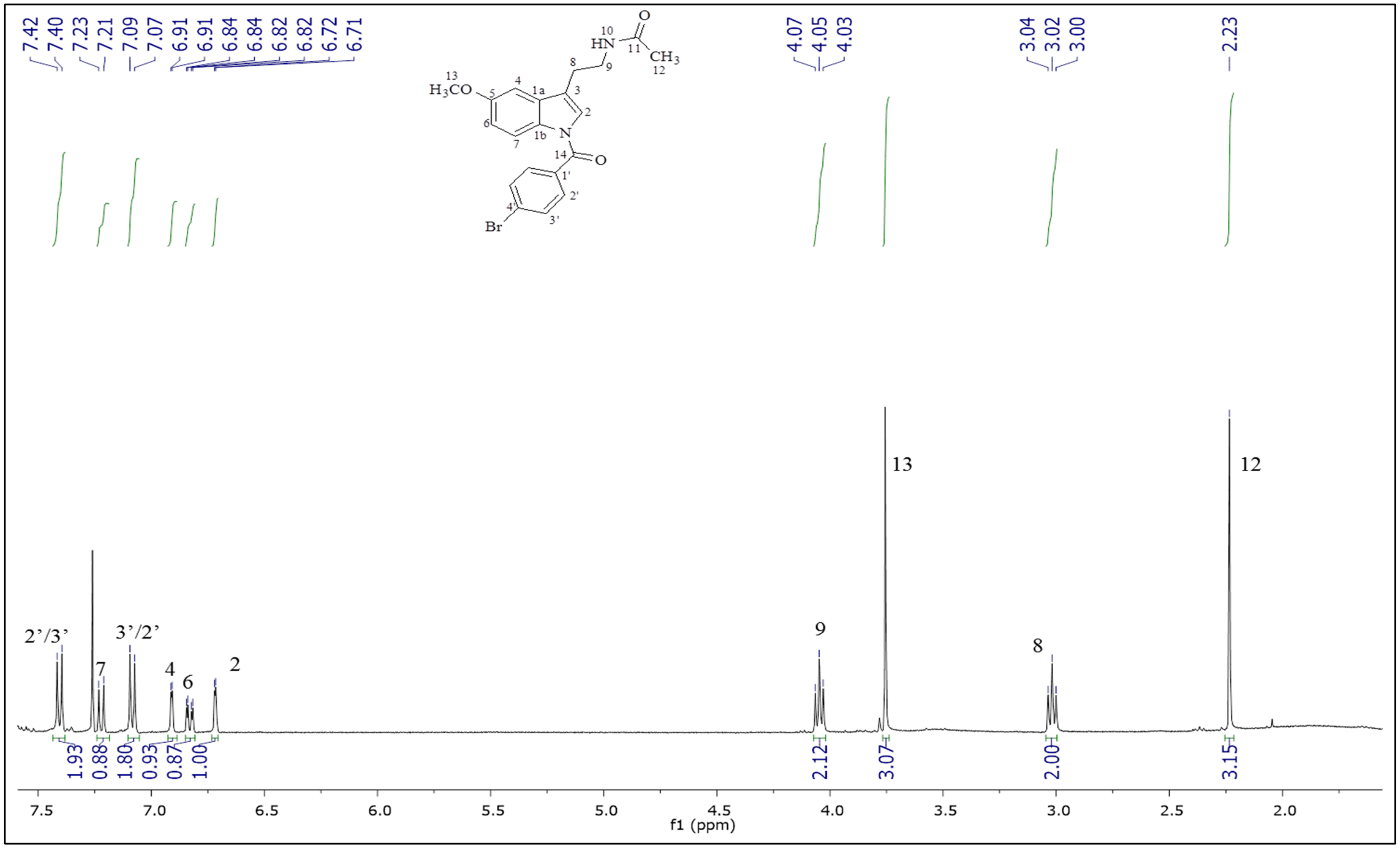

Figure S1. ^{1}H-NMR spectrum of BBM

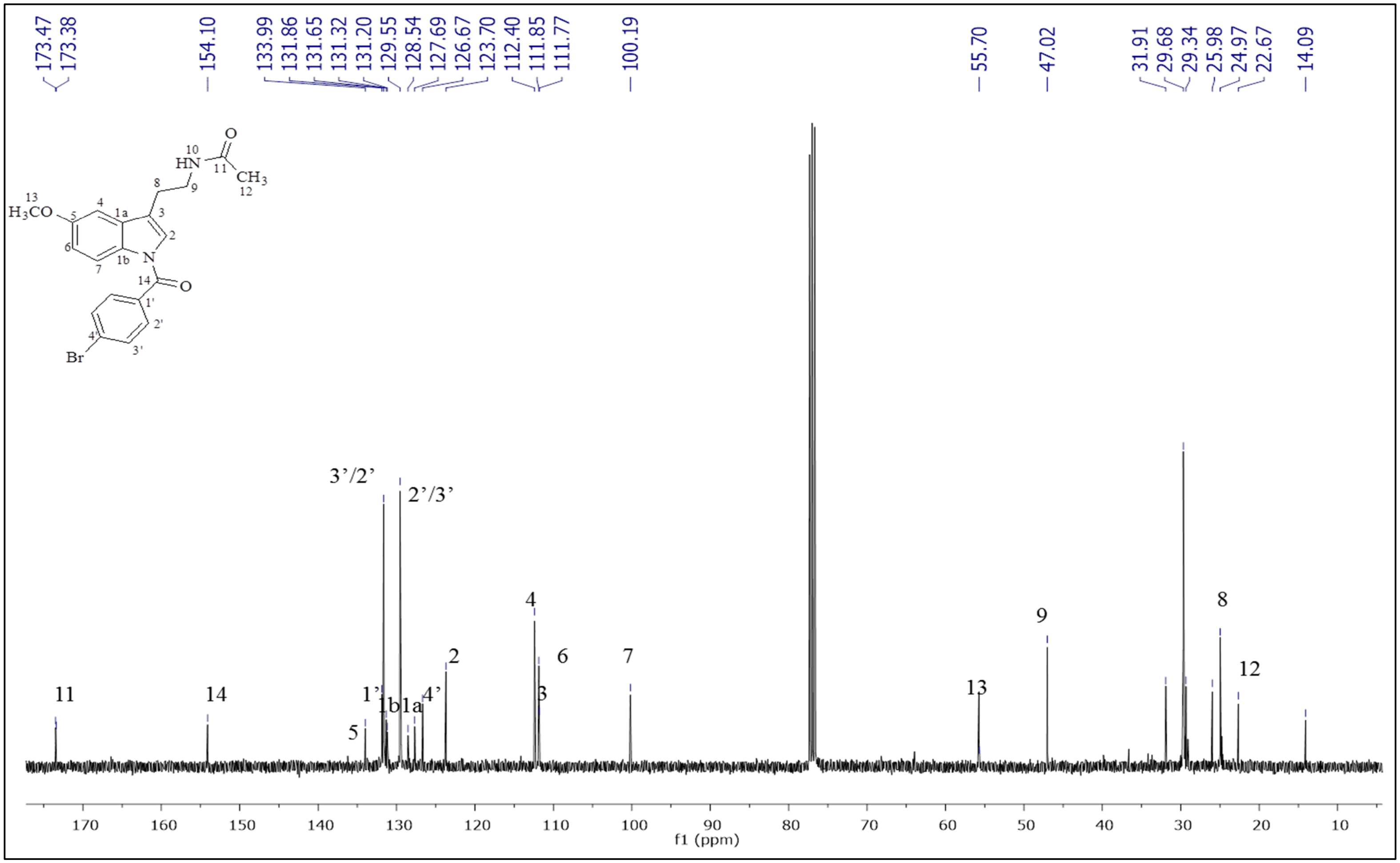

Figure S2. ^{13}C-NMR spectrum of BBM

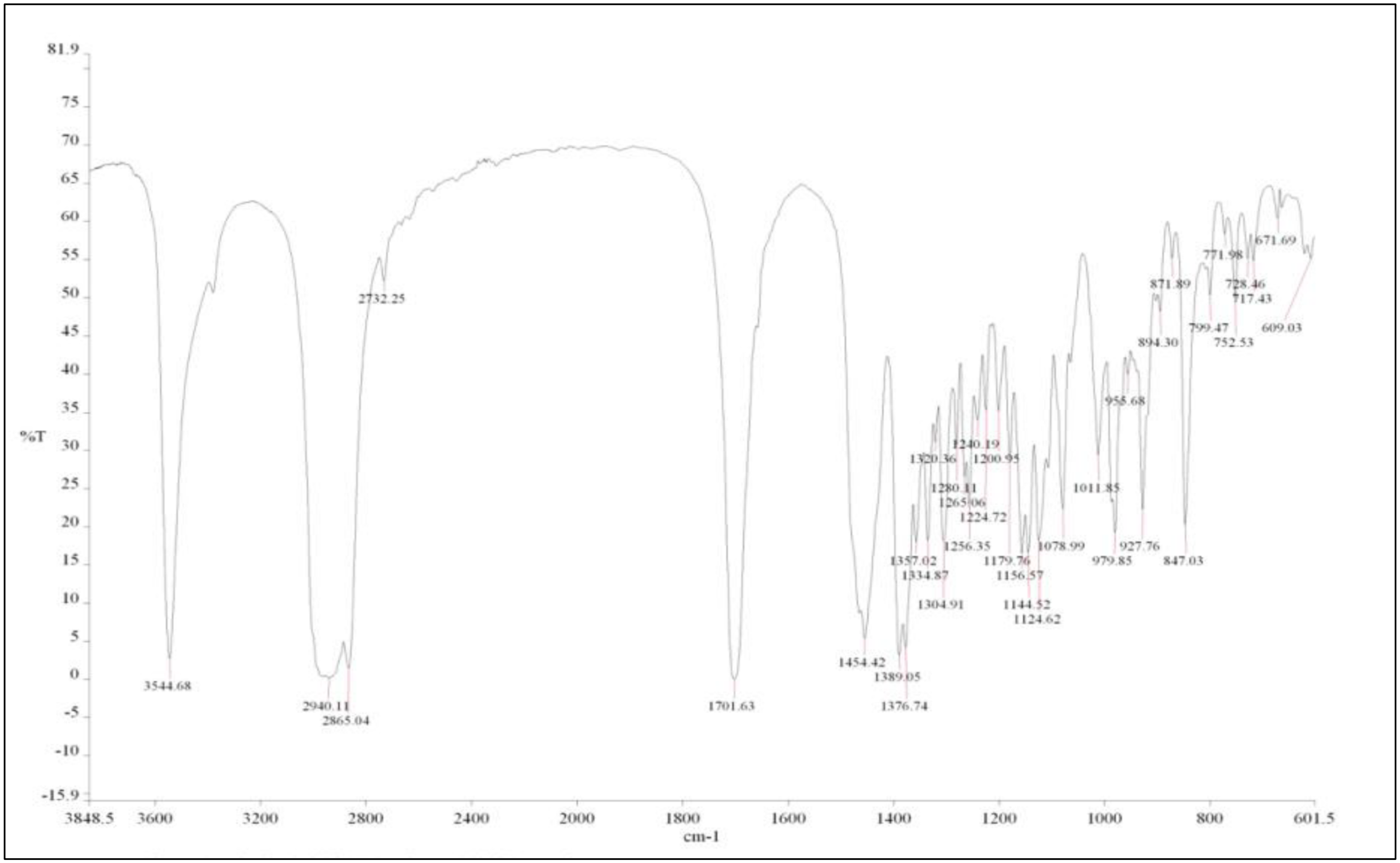

Figure S3. IR spectrum of BBM

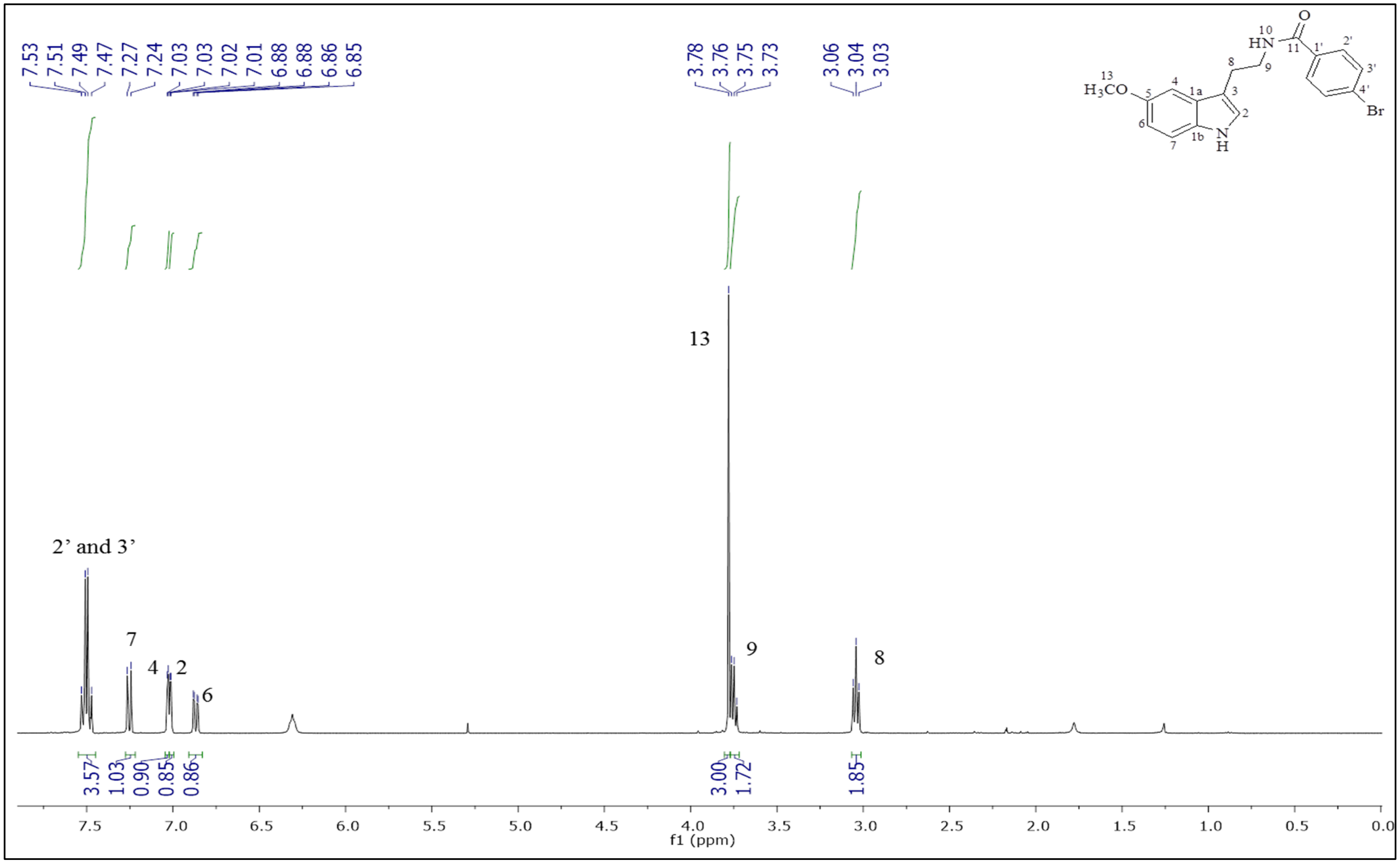

Figure S4. ^{1}H-NMR spectrum of EBM

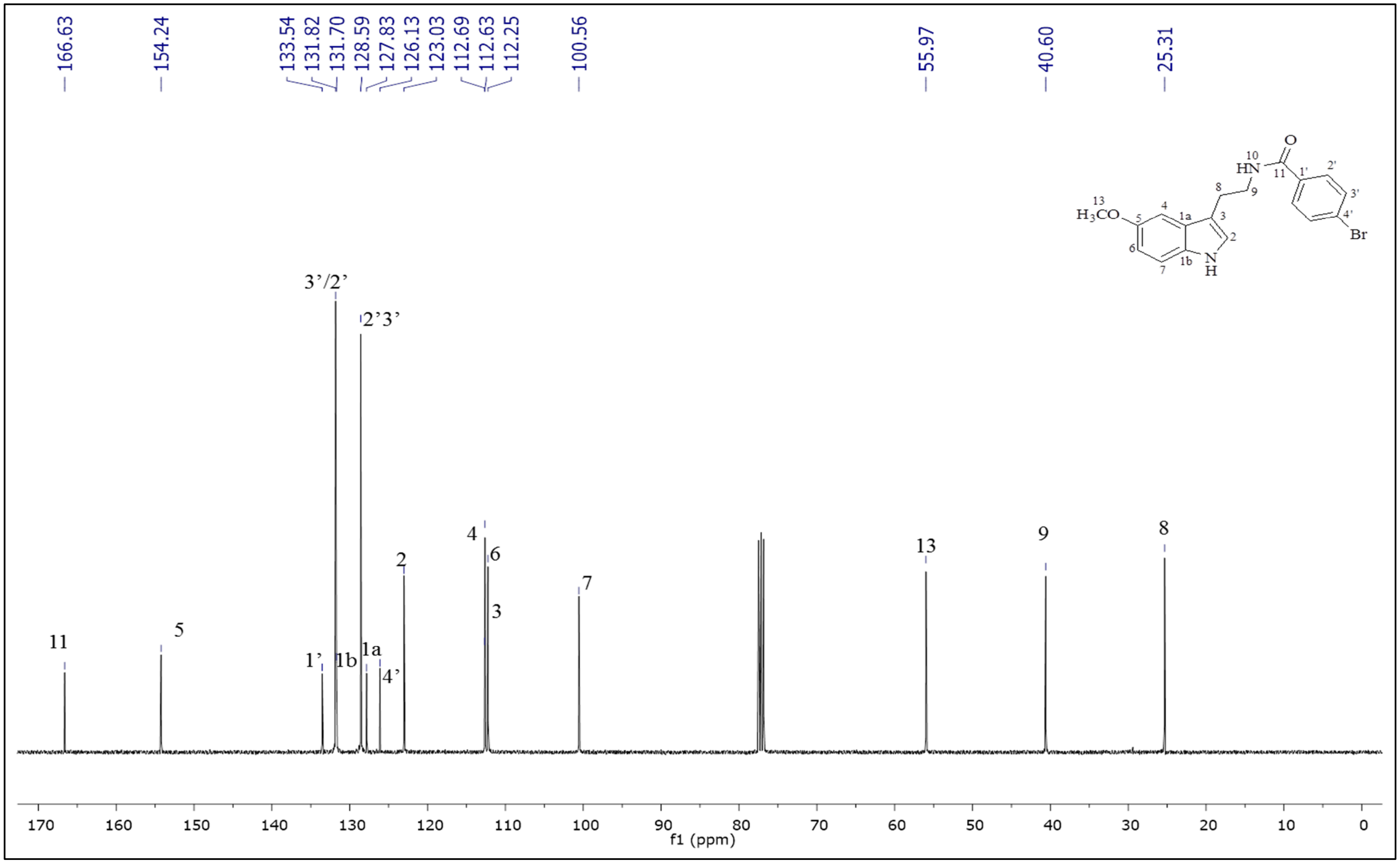

Figure S5. ^{13}C-NMR spectrum of EBM

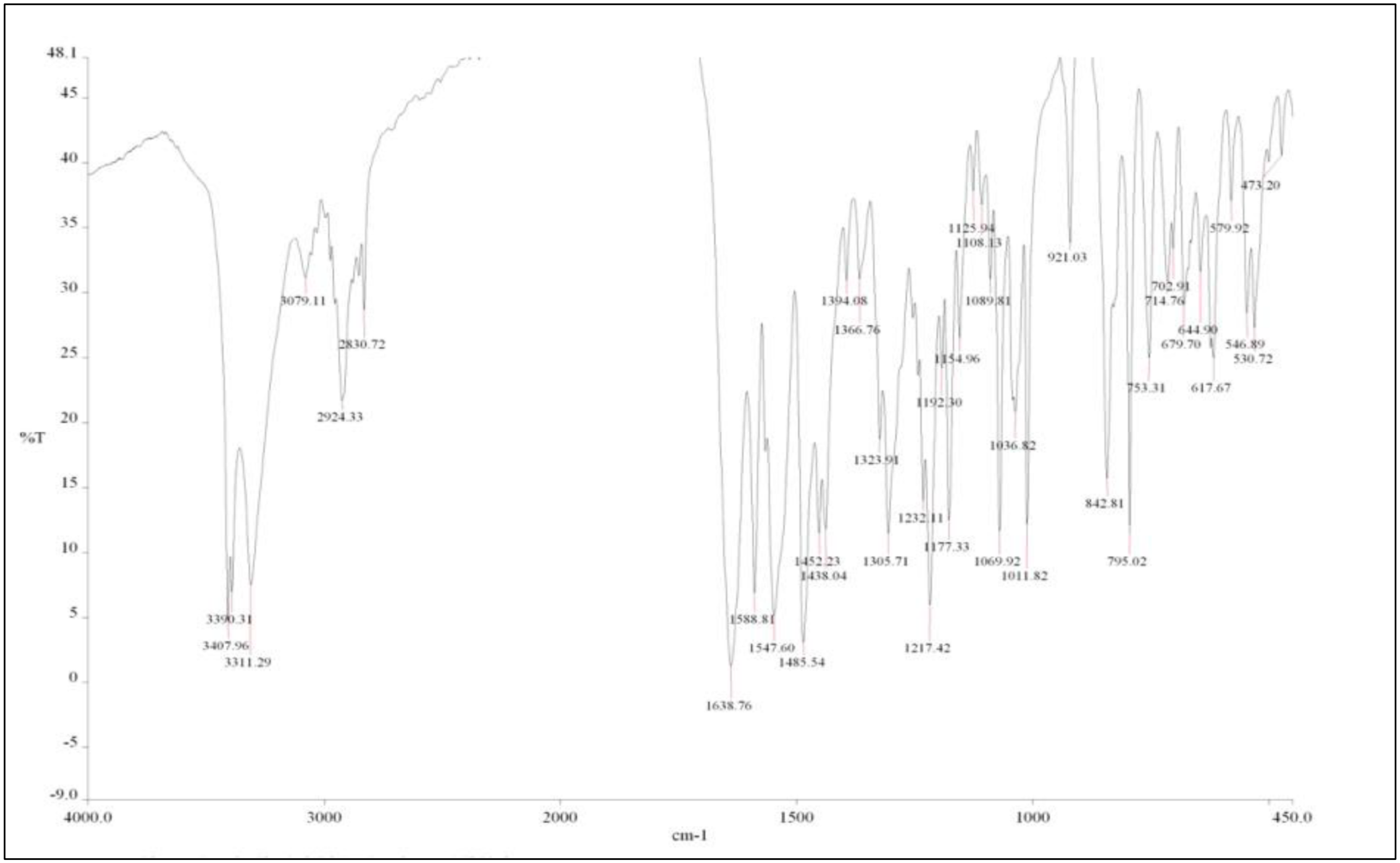

Figure S6. IR spectrum of EBM

Table S1. MW, Lipophilicity values in Octanol-water (Log $P_{o/w}$), topological polar surface area (TPSA), solubility parameter (log S), permeability coefficient (log K_p) predicted from Swiss ADME.

Parameters/ compounds	Melatonin	BBM	EBM
MW	232.28	415.28	373.24
Log $P_{o/w}$	1.83	3.63	3.65
TPSA (A^0)	54.12	60.33	54.12
Log S	-2.34	-4.76	-4.72
Log K_p	-6.59	-6.15	-5.78
